# Association Between Cribriform Architecture and Tertiary Gleason Pattern 5 in Prostate Cancer: A Cross-Sectional Study of Radical Prostatectomy Specimens

**DOI:** 10.3390/jcm15124637

**Published:** 2026-06-15

**Authors:** Sayeh Fattahi, Yetkin Tuac, Okan Argun, Bryce Thomsen, Alicia C. Smart, Fallon E. Chipidza, Jonathan E. Leeman, Mutlay Sayan

**Affiliations:** 1 Department of Radiation Oncology, Brigham and Women’s Hospital and Dana Farber Cancer Institute, Harvard Medical School, Boston, MA 02115, USA; 2 Department of Statistics, Ankara University, Ankara 06100, Türkiye

**Keywords:** prostate cancer, cribriform architecture, tertiary Gleason pattern 5, radical prostatectomy

## Abstract

**Background/Objectives:** Cribriform architecture is an adverse Gleason pattern 4 morphology associated with aggressive prostate cancer outcomes. Tertiary Gleason pattern 5, even as a minor component, may also identify tumors with higher-grade biology not fully captured by conventional Grade Group assignment. We examined whether cribriform architecture is associated with tertiary Gleason pattern 5 in patients undergoing radical prostatectomy. **Methods:** We performed a retrospective cross-sectional study of radical prostatectomy specimens from patients with prostate adenocarcinoma who underwent radical prostatectomy and had available clinicopathologic data. A centralized pathology review of digitized radical prostatectomy slides was used to assess cribriform architecture. Tertiary Gleason pattern 5 status was obtained from original pathology reports. Multivariable logistic regression was used to evaluate the association between cribriform architecture and tertiary Gleason pattern 5 after adjustment for age, preoperative prostate-specific antigen level, prostatectomy Gleason score, pathologic tumor stage, and margin status. **Results:** Among 303 patients, 47 (15.5%) had tertiary Gleason pattern 5. Cribriform architecture was more common in tumors with tertiary Gleason pattern 5 than in those without (70% vs. 21%; *p* < 0.001). On multivariable analysis, cribriform architecture remained independently associated with tertiary Gleason pattern 5 (adjusted odds ratio of 9.46; 95% confidence interval of 4.49–21.0; *p* < 0.001). The model demonstrated good discrimination, with an area under the receiver operating characteristic curve of 0.80. **Conclusions:** Cribriform architecture was strongly associated with tertiary Gleason pattern 5. These findings suggest that cribriform-positive tumors may be more likely to harbor minor high-grade pattern 5 components.

## 1. Introduction

Accurate grading remains central to risk stratification in localized prostate cancer, yet tumors within the same Gleason score or Grade Group can show substantial biological heterogeneity [1,2,3,4]. Beyond the primary and secondary Gleason patterns, additional adverse morphologic features may provide important prognostic information. Cribriform architecture, a Gleason pattern 4 morphology, has emerged as one of the most clinically meaningful adverse histologic features in prostate cancer and has been associated with higher rates of biochemical recurrence, metastasis, and prostate cancer–specific mortality [5,6,7,8,9,10,11,12,13,14,15,16]. Similarly, tertiary Gleason pattern 5, even when present as a minor component, has been linked to worse oncologic outcomes and may indicate tumors with more aggressive biology than suggested by the assigned Grade Group alone [17,18,19,20].

Although cribriform architecture and tertiary Gleason pattern 5 are both adverse histopathologic findings, their relationship has not been well characterized. This represents a primarily diagnostic and pathologic gap rather than a treatment-outcome question: it remains unclear whether cribriform architecture is associated with the concurrent presence of minor high-grade pattern 5 in prostatectomy specimens. This gap is clinically relevant because both features may refine risk within otherwise similar Gleason categories. Cribriform architecture represents an aggressive pattern 4 morphology, whereas tertiary Gleason pattern 5 reflects the presence of a small but high-grade component that is not fully captured by the standard Grade Group assignment [21,22,23,24,25]. Determining whether these features frequently coexist may provide insight into whether cribriform-positive tumors represent a morphologic phenotype associated with occult or underrecognized high-grade disease.

As such, we examined the association between cribriform architecture and tertiary Gleason pattern 5 in radical prostatectomy specimens. We hypothesized that cribriform-positive tumors are more likely to demonstrate tertiary Gleason pattern 5, independent of standard clinicopathologic factors. This cross-sectional study aimed to evaluate the association between two important adverse histopathologic features and provide additional context for the biological heterogeneity of prostate cancer beyond conventional Gleason grading.

## 2. Materials and Methods

### 2.1. Study Population

**Figure 1:**
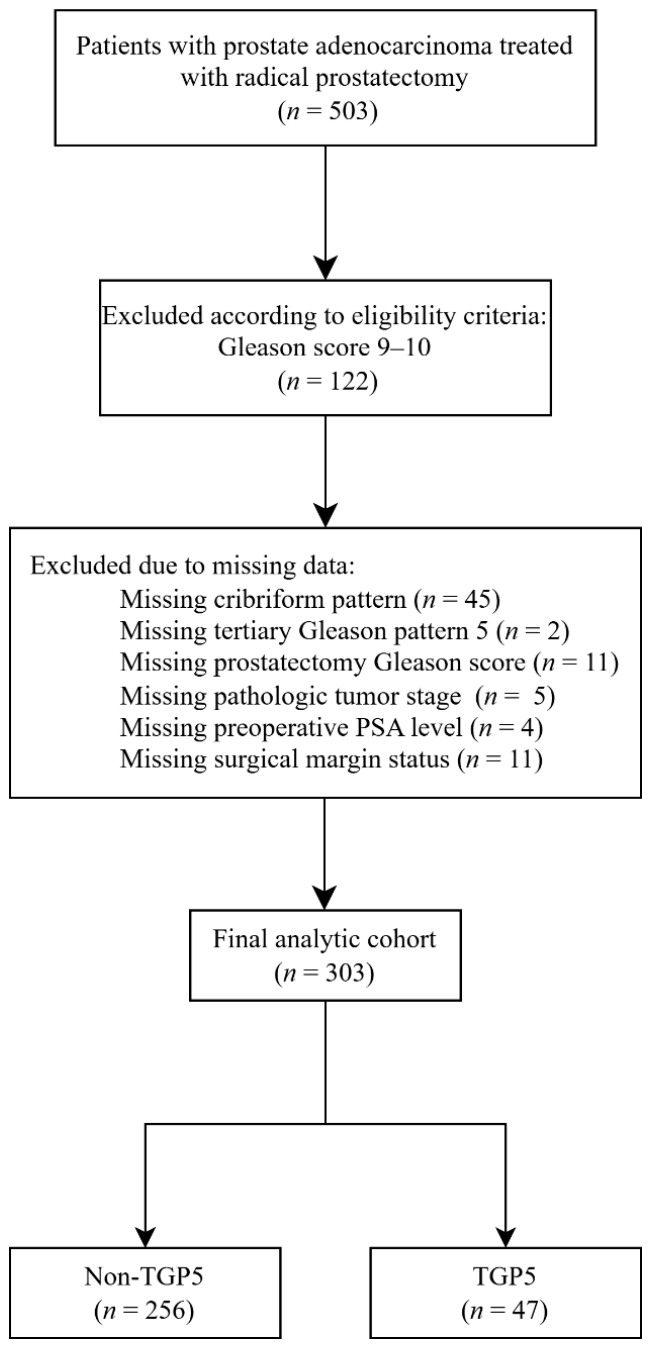
Flow diagram illustrating patient selection for the study. The source population included 503 radical prostatectomy cases. Gleason score 9–10 tumors were excluded as an eligibility criterion because tertiary Gleason pattern 5 was not applicable by definition in tumors where Gleason pattern 5 was incorporated into the primary or secondary Gleason score. The remaining 381 patients constituted the eligible study population, of whom 78 were excluded because of incomplete required clinicopathologic information, resulting in a final analytic cohort of 303 patients. Abbreviation: PSA, prostate-specific antigen; TGP5, tertiary Gleason pattern 5.

We performed a retrospective cross-sectional study of radical prostatectomy specimens from patients with prostate adenocarcinoma from The Cancer Genome Atlas (TCGA) prostate cancer dataset. The source population consisted of 503 patients with prostate adenocarcinoma who underwent radical prostatectomy between January 2000 and December 2023 (Figure 1). Patients with prostatectomy Gleason score 9–10 were excluded as an eligibility criterion because Gleason pattern 5 is incorporated into the primary or secondary Gleason score in these tumors rather than reported as a tertiary pattern. Therefore, tertiary Gleason pattern 5, as defined for this study, was not applicable to Gleason score 9–10 tumors. After exclusion of 122 patients with Gleason score 9–10 disease, 381 patients constituted the eligible study population. Patients were then excluded if key variables required for the analysis were missing, including prostatectomy Gleason score, pathologic tumor stage, preoperative prostate-specific antigen (PSA) level, prostatectomy margin status, or tertiary Gleason pattern 5 status. Among the 381 eligible patients, 78 were excluded because of incomplete required clinicopathologic information, including missing cribriform architecture status (*n* = 45), tertiary Gleason pattern 5 status (*n* = 2), prostatectomy Gleason score (*n* = 11), pathologic tumor stage (*n* = 5), preoperative PSA level (*n* = 4), and surgical margin status (*n* = 11). This resulted in a final analytic cohort of 303 patients. Complete-case analysis was used for the primary analysis.

A centralized pathology review of digitized radical prostatectomy slides performed as part of the TCGA pathology review process was used to identify cribriform architecture, as previously described [26,27]. The centralized review was conducted by three genitourinary pathologists using established morphologic criteria to determine the presence or absence of cribriform architecture on radical prostatectomy specimens. Consistent with prior TCGA pathology review methods, the assessment followed accepted histopathologic definitions of cribriform growth patterns, including distinction from intraductal carcinoma based on recognized morphologic features. However, intraductal carcinoma status was not captured as a separate variable in the TCGA dataset because the primary objective of the original pathology review was to determine the presence or absence of cribriform architecture; therefore, a standardized analysis of intraductal carcinoma was not possible in the present study. Tertiary Gleason pattern 5 status was obtained from the available original pathology reports within the TCGA dataset and was recorded as a binary variable, defined as present or absent.

This study used de-identified publicly available data and was conducted in accordance with the Declaration of Helsinki. Institutional review board exemption was obtained from our institution, and due to the use of de-identified data, the requirement for informed consent was waived.

### 2.2. Statistical Analysis

Baseline clinical and pathologic characteristics, stratified by the presence or absence of tertiary Gleason pattern 5, were summarized using descriptive statistics. Continuous variables, including age and preoperative PSA level, were reported as medians with interquartile ranges (IQR) and compared using the Wilcoxon rank-sum test [28]. Categorical variables, including cribriform architecture, prostatectomy Gleason score, pathologic tumor stage, margin status, adjuvant treatment, and perineural invasion, were summarized as frequencies and percentages and compared using Pearson’s chi-squared test or Fisher’s exact test [29], as appropriate.

The primary objective was to evaluate whether cribriform architecture was associated with tertiary Gleason pattern 5. Unadjusted associations between cribriform architecture and tertiary Gleason pattern 5 were evaluated using 2 × 2 contingency tables. The prevalence of tertiary Gleason pattern 5 was compared between cribriform-positive and cribriform-negative tumors, and crude odds ratios with 95% confidence intervals were calculated.

Multivariable logistic regression was then used to assess whether cribriform architecture was independently associated with tertiary Gleason pattern 5 after adjustment for prespecified clinicopathologic factors. The final model included age, preoperative PSA level, prostatectomy Gleason score, pathologic tumor stage, and prostatectomy margin status. These covariates were selected a priori based on their clinical relevance to prostate cancer aggressiveness and pathologic risk. The adjustment strategy was intended to evaluate whether the association between cribriform architecture and tertiary Gleason pattern 5 persisted after accounting for standard clinicopathologic factors available in routine prostatectomy assessment. Given the modest number of tertiary Gleason pattern 5 events (*n* = 47), the model was intentionally limited to prespecified clinically relevant variables, and no data-driven variable selection was performed. With 47 events and six model predictors, the events-per-variable ratio was approximately 7.8; therefore, more complex modeling was avoided. The area under the receiver operating characteristic curve was reported to describe model discrimination; Model performance and robustness were further assessed using the area under the ROC curve with 95% confidence interval, variance inflation factors for multicollinearity, the Hosmer–Lemeshow goodness-of-fit test for calibration, and sensitivity analyses using simpler adjustment models.

Adjusted odds ratios with 95% confidence intervals were reported for all predictors. Model discrimination was assessed using receiver operating characteristic curve analysis and quantified using the area under the curve [30]. All statistical tests were two-sided, and statistical significance was defined as *p* < 0.05. All analyses were performed using R software (version 4.2.3; R Foundation for Statistical Computing, Vienna, Austria).

## 3. Results

### 3.1. Baseline Clinical and Pathologic Characteristics

In total, 303 patients were included in our analysis, of whom 47 patients (15.5%) had tertiary Gleason pattern 5 and 256 patients (84.5%) did not. Baseline clinical and pathologic characteristics stratified by tertiary Gleason pattern 5 status are shown in Table 1. Age was similar between patients with and without tertiary Gleason pattern 5 (median, 62 years [IQR, 55–66 years] vs. 61 years [IQR, 55–66 years]; *p* = 0.600). Pre-radical prostatectomy PSA levels were numerically higher among patients with tertiary Gleason pattern 5, although this difference was not statistically significant (median, 8 ng/mL [IQR, 5–15 ng/mL] vs. 7 ng/mL [IQR, 5–10 ng/mL]; *p* = 0.110).

Patients with tertiary Gleason pattern 5 were more likely to have adverse pathologic features. Compared with patients without tertiary Gleason pattern 5, those with tertiary Gleason pattern 5 were more likely to have extraprostatic disease, defined as pathologic stage ≥ pT3a (74% vs. 48%; *p* < 0.001). The distribution of prostatectomy Gleason score did not differ significantly between groups (Gleason score 8: 21% vs. 17%; *p* = 0.500). Prostatectomy margin status, adjuvant treatment use, and perineural invasion were not significantly different between groups.

Cribriform architecture was substantially more common among tumors with tertiary Gleason pattern 5 compared with those without tertiary Gleason pattern 5. Among patients with tertiary Gleason pattern 5, 33 of 47 tumors (70%) demonstrated cribriform architecture, compared with 54 of 256 tumors (21%) among patients without tertiary Gleason pattern 5 (*p* < 0.001). In unadjusted analysis, tertiary Gleason pattern 5 was present in 33 of 87 cribriform-positive tumors (37.9%) compared with 14 of 216 cribriform-negative tumors (6.5%), corresponding to a crude odds ratio of 8.80 (95% CI, 4.38–17.68; *p* < 0.001).

Baseline clinical and pathologic characteristics were compared between patients included in the final analytic cohort and those excluded due to missing data. This comparison was restricted to the 381 otherwise eligible patients after exclusion of Gleason score 9–10 tumors, because Gleason score 9–10 disease was an eligibility exclusion rather than a missing-data exclusion. As shown in Supplementary Table S1, no statistically significant differences were observed between the 303 included patients and the 78 otherwise eligible patients excluded because of incomplete required clinicopathologic information two groups for the evaluated characteristics.

### 3.2. Multivariable Analysis for Predictors of Tertiary Gleason Pattern 5

Multivariable logistic regression was performed to evaluate whether cribriform architecture was independently associated with tertiary Gleason pattern 5 after adjustment for age, pre-radical prostatectomy PSA, prostatectomy Gleason score, pathologic tumor stage, and prostatectomy margin status. As shown in Table 2, cribriform architecture remained strongly associated with tertiary Gleason pattern 5 on multivariable analysis (adjusted odds ratio [aOR], 9.46; 95% CI, 4.49–21.0; *p* < 0.001).

Among the other covariates included in the model, pathologic tumor stage ≥ pT3a showed a trend toward association with tertiary Gleason pattern 5 but did not reach statistical significance (aOR, 2.14; 95% CI, 0.98–4.86; *p* = 0.061). Age, pre-radical prostatectomy PSA, prostatectomy Gleason score 8, and positive surgical margin status were not significantly associated with tertiary Gleason pattern 5 after adjustment.

**Figure 2:**
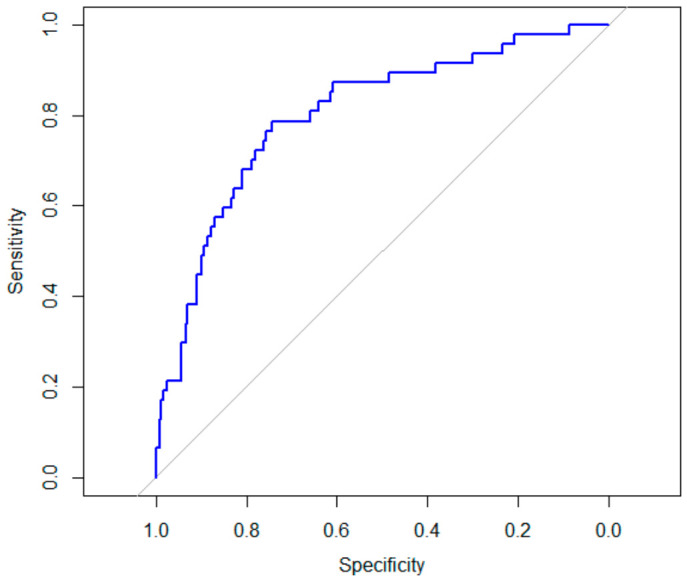
Receiver operating characteristic (ROC) curve for predicting tertiary Gleason pattern 5. The blue line represents the ROC curve, and the gray diagonal line represents the no-discrimination reference line.

The multivariable logistic regression model was interpreted as an adjusted association analysis. Cribriform architecture remained strongly associated with tertiary Gleason pattern 5 after adjustment for clinicopathological covariates. Additional model diagnostics and sensitivity analyses are presented in Supplementary Table S2. The model showed acceptable discrimination (AUC, 0.802; 95% CI, 0.733–0.872), no substantial multicollinearity (maximum VIF, 1.191), and acceptable calibration by the Hosmer–Lemeshow test (*p* = 0.752). In simpler adjustment models, the association between cribriform architecture and tertiary Gleason pattern 5 remained directionally consistent. The ROC curve assessing the discriminatory ability of the model to predict tertiary Gleason pattern 5 is presented in Figure 2.

## 4. Discussion

In this retrospective cross-sectional study of radical prostatectomy specimens from patients with prostate cancer treated with radical prostatectomy, we evaluated the association between cribriform architecture and tertiary Gleason pattern 5, two adverse histopathologic features that may refine risk beyond conventional Grade Group assignment. We found that cribriform architecture was substantially more common in tumors with tertiary Gleason pattern 5 than in those without tertiary pattern 5. Importantly, this association remained strong after adjustment for age, preoperative PSA, prostatectomy Gleason score, pathologic tumor stage, and margin status. These findings suggest that cribriform morphology may identify a subset of prostate cancers with a higher likelihood of harboring high-grade pattern 5 disease, supporting the concept that cribriform-positive tumors represent a biologically aggressive phenotype not fully captured by standard Gleason grading alone. However, because this study was designed to evaluate a pathologic association and did not include long-term clinical outcomes, these findings should not be interpreted as establishing the prognostic impact of the combined presence of cribriform architecture and tertiary Gleason pattern 5.

Cribriform architecture has increasingly been recognized as one of the most clinically meaningful Gleason pattern 4 morphologies. Prior studies have shown that cribriform-positive tumors are associated with adverse pathologic features and worse oncologic outcomes, including higher rates of biochemical recurrence, metastasis, and prostate cancer–specific mortality [5,6,7,8,9,10,11,12,13,14,15,16]. Similarly, tertiary Gleason pattern 5 has been associated with worse outcomes, even when present as a small component of the tumor [17,18,19,20]. The current ISUP Grade Group system does not formally incorporate tertiary pattern 5, but its presence may identify patients whose disease behaves more aggressively than expected based on the assigned primary and secondary Gleason patterns [20,21,22,23].

The observed association between cribriform architecture and tertiary Gleason pattern 5 may have important biological implications. Cribriform morphology is classified as Gleason pattern 4 [24], but its clinical behavior often appears closer to higher-grade disease than to other pattern 4 morphologies. One possible explanation is that cribriform-positive tumors may be more likely to co-occur with poorly differentiated pattern 5 components. In this context, tertiary Gleason pattern 5 may represent a histologic marker of intratumoral grade heterogeneity rather than evidence of temporal histologic progression. The strong association observed in this study supports the hypothesis that cribriform architecture is not merely another pattern 4 subtype, but rather a morphologic feature that may indicate a broader aggressive histologic program. However, because cribriform architecture and tertiary Gleason pattern 5 were assessed at the same pathologic time point, this study cannot determine whether one feature precedes or gives rise to the other.

These findings may also help explain why patients with cribriform-positive disease experience worse outcomes even within the same Gleason score or Grade Group. Conventional Gleason grading is based on the most prevalent and second most prevalent patterns [22], and therefore small amounts of pattern 5 may not be reflected in the final Grade Group in radical prostatectomy specimens. If cribriform architecture is associated with tertiary pattern 5, then cribriform-positive status may partly capture the presence of high-grade tumor elements that are otherwise underrepresented in standard grade assignment. This may be particularly relevant in Grade Group 2 or 3 tumors, where the presence of cribriform architecture or tertiary pattern 5 could identify patients who are at higher risk than their Grade Group alone would suggest.

From a clinical perspective, these findings support careful reporting and interpretation of both cribriform architecture and tertiary Gleason pattern 5 in prostatectomy specimens. Although this study does not directly evaluate treatment outcomes, the coexistence of these features may identify patients who warrant further study in cohorts with long-term follow-up. This may be especially relevant when counseling patients whose disease appears intermediate-risk by conventional clinicopathologic criteria [31], but contains adverse morphologic features associated with aggressive biology. As pathology reporting evolves, incorporating these features into risk stratification models may improve the ability to distinguish biologically favorable from unfavorable disease within the same Gleason score category. Future studies should also evaluate whether combining cribriform architecture and tertiary Gleason pattern 5 with molecular features or genomic classifiers can further refine risk stratification; however, whether such combined assessment should influence treatment selection requires prospective validation with clinical outcome data.

The potential relevance of these findings may extend to diagnostic biopsy interpretation as well. Cribriform architecture can often be identified on biopsy and is increasingly recognized as a feature that may influence active surveillance eligibility and treatment decision-making [16]. In contrast, tertiary Gleason pattern 5 is generally a prostatectomy-based concept [22], while on biopsy the highest-grade component is typically incorporated into the Gleason score. Therefore, if cribriform morphology on biopsy is associated with a greater likelihood of minor pattern 5 at prostatectomy, its presence may signal unsampled high-grade disease. Future studies comparing biopsy cribriform status with prostatectomy tertiary Gleason pattern 5 could help clarify whether cribriform architecture can serve as an early marker for occult high-grade components.

Despite these findings, this study does have limitations. First, the retrospective nature of this study limits causal inference and introduces the possibility of selection bias. Accordingly, the present study should be interpreted as an exploratory cross-sectional association study of two pathologic features rather than evidence that cribriform architecture causes tertiary Gleason pattern 5, precedes tertiary Gleason pattern 5, or that their coexistence independently predicts clinical outcomes. Second, tertiary Gleason pattern 5 status was obtained from available original pathology reports within the TCGA dataset rather than from a dedicated centralized rereview for this specific feature, which may introduce variability in reporting and possible underreporting. Third, although cribriform architecture was assessed through a centralized pathology review, the distinction between invasive cribriform carcinoma versus intraductal carcinoma was not the primary focus of this analysis and could not be standardized using the available data. In addition, information regarding reviewer blinding and formal interobserver reproducibility assessment was not available. Fourth, the cohort included surgically treated patients from TCGA, a selected research cohort with available digitized pathology and genomic data, and may not be fully generalizable to patients managed with radiation therapy, active surveillance, or contemporary molecularly guided treatment strategies. Fifth, the number of patients with tertiary Gleason pattern 5 was modest, which may limit the stability of multivariable estimates despite the use of a prespecified parsimonious model. Furthermore, because variables such as Gleason score and pathologic stage are closely related to tumor aggressiveness, the adjusted model may represent a conservative approach and could be affected by partial overadjustment or collinearity, although no substantial multicollinearity was observed by variance inflation factor assessment. Finally, clinical outcomes were not evaluated in the present analysis, and therefore the prognostic implications of combined cribriform architecture and tertiary Gleason pattern 5 require validation in cohorts with long-term follow-up.

## 5. Conclusions

Cribriform architecture was strongly and independently associated with tertiary Gleason pattern 5 in this cross-sectional study of radical prostatectomy specimens. These findings provide additional pathologic context for the aggressive clinical behavior previously reported in cribriform-positive prostate cancer and suggest that cribriform morphology may identify tumors more likely to harbor minor high-grade Gleason pattern 5 components. Prospective studies with standardized pathology review and long-term clinical outcomes are warranted to determine whether combined assessment of cribriform architecture and tertiary Gleason pattern 5 can improve postoperative risk stratification or inform treatment selection.

## Figures and Tables

**Table 1:** Baseline clinical and pathologic factors stratified by tertiary Gleason pattern 5.

	Non-TGP5 (*n* = 256)	TGP5 (*n* = 47)	*p*
Age (years), median (IQR)	61 (55, 66)	62 (55, 66)	0.600
Pre-RP PSA, ng/mL, median (IQR)	7 (5, 10)	8 (5, 15)	0.110
Prostatectomy Gleason score, No. (%)			0.500
≤7	212 (83%)	37 (79%)	
8	44 (17%)	10 (21%)	
Prostatectomy tumor stage, No. (%)			<0.001
T2	134 (52%)	12 (26%)	
≥pT3a	122 (48%)	35 (74%)	
Prostatectomy margin status, No. (%)			0.700
Negative	191 (75%)	34 (72%)	
Positive	65 (25%)	13 (28%)	
Adjuvant treatment, No. (%)			0.300
No	240 (94%)	42 (89%)	
Yes	16 (6%)	5 (11%)	
PNI, No. (%)			0.400
No	63 (25%)	9 (19%)	
Yes	193 (75%)	38 (81%)	
Cribriform Architecture, No. (%)			<0.001
No	202 (79%)	14 (30%)	
Yes	54 (21%)	33 (70%)	

Abbreviations: TGP5, tertiary Gleason pattern 5; RP, radical prostatectomy; PSA, prostate-specific antigen; PNI, perineural invasion; IQR, interquartile range.

**Table 2:** Multivariable logistic regression analysis for predictors of tertiary Gleason pattern 5.

	aOR	95% CI	*p*
Cribriform Architecture			
No	Reference	Reference	
Yes	9.46	4.49–21.0	<0.001
Age (years)	1.00	0.95–1.06	0.900
Pre-RP PSA, ng/mL,	1.00	0.97–1.03	0.800
Prostatectomy Gleason score			
≤7	Reference	Reference	
8	0.51	0.20–1.22	0.150
Prostatectomy tumor stage			
T2	Reference	Reference	
≥pT3a	2.14	0.98–4.86	0.061
Prostatectomy Margin status			
Negative	Reference	Reference	
Positive	1.64	0.71–3.71	0.200

Abbreviations: CI, confidence interval; aOR, adjusted odds ratio; RP, radical prostatectomy; PSA, prostate-specific antigen.

## Data Availability

The original contributions presented in this study are included in the article. Further inquiries can be directed to the corresponding author.

## Supplementary Materials

The following supporting information can be downloaded at: https://www.mdpi.com/article/10.3390/jcm15124637/s1, Supplementary Table S1. Comparison of baseline clinicopathological characteristics between the final analytic cohort and otherwise eligible patients excluded due to missing data. Supplementary Table S2. Model diagnostics and sensitivity analyses for the association between cribriform architecture and tertiary Gleason pattern 5.

## Abbreviations

The following abbreviations are used in this manuscript:

aOR	Adjusted odds ratio
AUC	Area under the curve
CI	Confidence interval
IQR	Interquartile range
PSA	Prostate-specific antigen
PNI	Perineural invasion
ROC	Receiver operating characteristic
RP	Radical prostatectomy
TCGA	The Cancer Genome Atlas
