# Supplementary material for: Association Between Cribriform Architecture and Tertiary Gleason Pattern 5 in Prostate Cancer: A Cross-Sectional Study of Radical Prostatectomy Specimens
Source: J Clin Med. 2026 Jun 15;15(12):4637. doi: 10.3390/jcm15124637 (PMC13302233; doi:10.3390/jcm15124637)
Supplement: Supplementary file 1 [file jcm-15-04637-s001.zip › jcm-4320375-supplementary.pdf]

Supplementary Table S1. Comparison of baseline clinicopathological characteristics between the final analytic cohort and otherwise eligible patients excluded due to missing data

	**Final analytic cohort (n = 303)**	**Excluded due to missing data (n = 78)**	**p**
Age (years), median (IQR)	61 (55, 66)	61 (56, 66)	0.729
Pre-RP PSA, ng/mL, median (IQR)	7 (5, 10)	7 (5, 11)	0.986
Prostatectomy Gleason score, No. (%)			0.111
7	249 (82%)	45 (73%)	
8	54 (18%)	17 (27%)	
Prostatectomy tumor stage, No. (%)			0.790
T2	146 (48%)	35 (51%)	
≥pT3a	157 (52%)	34 (49%)	
Prostatectomy margin status, No. (%)			0.520
Negative	225 (74%)	49 (79%)	
Positive	78 (26%)	13 (21%)	
Adjuvant treatment, No. (%)			0.802
No	282 (93%)	69 (92%)	
Yes	21 (6.9%)	6 (8.0%)	
PNI, No. (%)			0.242
No	72 (10%)	24 (18%)	
Yes	231 (90%)	54 (82%)	
Cribriform Architecture, No. (%)			0.687
No	216 (71%)	25 (76%)	
Yes	87 (29%)	8 (24%)	

Abbreviations: TGP5, tertiary Gleason pattern 5; RP, radical prostatectomy; PSA, prostate-specific antigen; PNI, perineural invasion; IQR, interquartile range.

Supplementary Table S2. Model diagnostics and sensitivity analyses for the association between cribriform architecture and tertiary Gleason pattern 5

Analysis	Result	P
Number of tertiary Gleason pattern 5 cases	47	—
Events per variable	7.8	—
AUC	0.802 (95% CI, 0.733–0.872)	—
Maximum variance inflation factor	1.191	—
Hosmer–Lemeshow goodness-of-fit test	Acceptable calibration	0.752
Unadjusted model: cribriform architecture	OR 8.82 (95% CI, 4.49–18.13)	<0.001
Adjusted for Gleason group and tumor stage	OR 8.37 (95% CI, 4.12–17.78)	<0.001
Adjusted for age and PSA	OR 8.66 (95% CI, 4.40–17.87)	<0.001
Fully adjusted model	OR 9.46 (95% CI, 4.50–21.05)	<0.001

Abbreviations: AUC: area under the receiver operating characteristic curve; CI: confidence interval; OR: odds ratio; PSA: prostate-specific antigen; VIF: variance inflation factor. Odds ratios refer to the association between cribriform architecture and tertiary Gleason pattern 5. The fully adjusted model included age, preoperative PSA, prostatectomy Gleason group, pathological tumor stage, and surgical margin status as adjustment covariates.
